# Supplementary material for: Effects of Community Water Fluoridation on Dental Caries Disparities in Adolescents
Source: Int J Environ Res Public Health. 2020 Mar 19;17(6):2020. doi: 10.3390/ijerph17062020 (PMC7175225; doi:10.3390/ijerph17062020)
Supplement: Supplementary file 1 [file ijerph-17-02020-s001.pdf]

Supplementary Material: Determining residential mobility and fluoride exposure from community drinking water in the North Carolina School Oral Health Survey

Assessment of Residential History

The parents or guardians were asked to provide the lifetime residential history of their child via a self-completed questionnaire in English or Spanish. They were asked questions on the current place of residence, and if it was not the only place in which the child had lived during his or her lifetime, then information on other residences, where the stay was at least two years long, were requested. For the current residence, the respondents were asked to estimate the amount of drinking water they usually consumed from three water sources (home well or spring, bottled water, city or community water system) using the options "All," "Most but not all," "Some," "None," or "Not sure," and asked the name of the water company, if applicable. For the past resident, they were also asked about the primary drinking water source (community water system, well water, unknown) and the name of the water company, if applicable, along with the number of years that the child had lived in that location.

Data Management and Assessment of Fluoride Status of Community Water System

Dental public health hygienists employed by public health agencies in North Carolina coded the fluoridation status of the current and previous community water systems listed in the completed questionnaires. They were responsible for conducting school-based oral health programs in the sampled schools and, hence, were familiar with the water systems and fluoridation status of the schools included in the survey. They were trained in coding and were provided information from the State Health Department and Centers for Disease Control and Prevention on the fluoridation status of community water systems in North Carolina. They also relied on local resources that could verify the fluoridation status, water distribution patterns, and personal knowledge of the fluoridation status of the communities in their jurisdiction. The water sources were coded according to the following fluoride status codes:

Table S1. Community Water Fluoride Status Codes Assigned by Project Field Staff.

Fluoride Status Code	Description of Fluoride Status Code
o	community water system, optimally adjusted (0.7–1.2 ppm)
m	community water system, mixed (system has suboptimal fluoride level and purchases water from another system on an as-needed basis)
na	community water system, naturally above optimal fluoride level (>1.2 ppm)
no	community water system, naturally optimal fluoride level (0.7–1.2 ppm)
nb	community water system, naturally below optimal fluoride level (<0.7 ppm)
x	community water system, no fluoride
missing	community water system not given

Source of Drinking Water

The parents' answers on the current water sources were divided into three major categories:

- City: The main water source was the community water system of the city. For example, the answers included "All" or "Most but not all" city water, or included "Some" city water but not "All," or "Most but not all" community water system.
- Well: The main water source was a well. For example, the answers included "All" or "Most but not all" well water or "some" well water but not "some" city water.
- Do not know (Dnk): We or they did not know the fluoridation level of their water source. This group included those whose main water source was bottled water and who did not give us enough information to identify the fluoride status of their drinking water source. Both the "Not logical" answer and "They do not know" answer were included in this category. "Not logical" represented any of the following combinations: "All" occurred more than once, "All" occurred once and "Most but not all" occurred one or more times, "Most but not all" occurred two or more times, "None" occurred at least once and the only other answers are missing, and "Some" occurred once with additional answers being only "None" or missing. "They do not know" set if "All" occurs zero times and "most but not all" occurs zero times and "some" occurs one or zero times and "Do not know" occurred one or more times.

The categorization of the current water sources is shown below:

Table S2. Categorization of Current Water Sources.

Detailed Category	Home Well or Spring	Bottled Water	City or Community Water System	Category
City only	4, 5, missing	4, 5, missing	1, 2	City
City, some bottle	4, 5, missing	3	1, 2	City
City, some well	3	4, 5, missing	1, 2	City
City, some bottle, some well	3	3	1,2	City
Well only	1, 2	4, 5, missing	4, 5, missing	Well
Well, some bottle	1, 2	3	4, 5, missing	Well
Well, some city	1, 2	4, 5, missing	3	Well
Well, some bottle, some city	1, 2	3	3	City
Bottle only	4, 5, missing	1, 2	4, 5, missing	Dnk
Bottle, some city	4, 5, missing	1, 2	3	City
Bottle, some well	3	1, 2	4, 5, missing	Well
Bottle, some city, some well	3	1, 2	3	City
Some city, some bottle	4, 5, missing	3	3	City
Some well, some bottle	3	3	4, 5, missing	Well
Some city, some well	3	4, 5, missing	3	City
Some city, some well, some bottle	3	3	3	City
Missing	missing	missing	missing	Dnk
Not logical	see above	see above	see above	Dnk
They do not know	see above	see above	see above	Dnk

"All" (1), "Most but not all" (2), "Some" (3), "None" (4), or "Not sure" (5). Dnk: Do not know.

Fluoride Status of Water Source at Current Residency

The categories of current water sources were cross-tabulated with the community water fluoride status codes.

Table S3. Responses by Water Source and Community Water Fluoride Status at Current Residence.

Water Source Category	Community water fluoride status codes							
	**x**	**o**	**nb**	**no**	**na**	**m**	**missing**	**Total**
**City**	248	2639	176	71	10	28	79	3251
**Well**	130	54	6	1	0	2	1454	1647
**Dnk**	58	387	31	7	3	3	574	1063
**Total**	436	3080	213	79	13	33	2107	5961

All samples included (age 5–19 years).

Transformation of Data on Current Water Sources

The major water source categories and fluoride status codes were combined into a single fluoride exposure code as given below:

Table S4. Developing Fluoride Exposure Code.

Major Water Category	Community Water Fluoride Status	Meaning of Fluoride Exposure Code	Fluoride Exposure Code
City	o	community water system, optimally adjusted	o
City	m	community water system, mixed	m
City	na	community water system, naturally above optimal fluoride level	na
City	no	community water system, naturally at optimal fluoride level	no
City	nb	community water system, naturally below optimal fluoride level	nb
City	x	community water system, no fluoride	x
City	missing	major water category is "city" and fluoride status codes is missing	dk (do not know)
Well	Not appl.	well	w
Dnk	Not appl.	do not know	dk

Transformation of Data on Past Water Sources

The parents were also asked whether their child had always lived at his/her current address. The answer choices were "Yes," "No," and "Not sure/Do not know." If the child always lived at the current address, then only the current data of fluoride exposure status were used and applied to the all their past statuses. If they had lived at other residences, then the information from the residential history and previous primary water sources was used to determine past status of fluoride exposure. The same fluoride exposure codes explained above were applied. The "dk" code was used to represent the response when the parents did not know the water source or the name of the community water system or they left it blank and review of forms by the local dental hygienists could not supply the correct information.

Analysis of Lifetime Exposure to Water Fluoridation

A series of 20 fluoride exposure variables was used to summarize the survey responses and characterize fluoride exposure for each year of the subject's life up to the exam date. At the exam date, all subjects were below 20 years; therefore, there were 20 members in the series, with each

member corresponding to the age of the subject. For example, if the fluoride status code for a subject's residence was "nb" and the subject was nine years old on the exam day, then the fluoride exposure series would look like this:

florExp0="nb", florExp1="nb", florExp2="nb", florExp3="nb", florExp4="nb", florExp5="nb", florExp6="nb", florExp7="nb", florExp8="nb", florExp9="nb", florExp10=missing, florExp11=missing, florExp12=missing, florExp13=missing, florExp14=missing, florExp15=missing, florExp16=missing, florExp17=missing, florExp18=missing, florExp19=missing,

where the name "florExp" is the first part of the SAS program variable name containing the fluoride exposure series and the digits following the characters "florExp" represent the age of the subject. The members in the series that were greater than a given subject's age were assigned missing values. The members that were equal to or less than a given subject's age were assigned one of the codes for the different categories of fluoride exposure (Table S4).

Some examples of the scheme used to assign codes to each of the 20 data elements during data management are given below:

Example 1:

A subject aged 15 years, on his/her classroom exam day, with current status data indicating a fluoride exposure code of "na". Further, this subject had three rows of data on previous residences as follows:

from	to	water	fl
3	5	city	o
7	9	well	
9	13	city	nb

The fluoride exposure series for this subject would be as follows:
florExp0=dk, florExp1=dk, florExp2=dk, florExp3=o, florExp4=o, florExp5=o, florExp6=dk, florExp7=w, florExp8=w, florExp9=w, florExp10=nb, florExp11=nb, florExp12=nb, florExp13=nb, florExp14=na, florExp15=na, florExp16=missing, florExp17= missing, florExp18= missing, florExp19= missing

Example 2:

A subject aged 12 years, on his/her classroom exam day, with current drinking water data indicating a fluoride exposure code of "no". Further, with three rows of data in the water history table, as follows:

from	to	water	fl
3	5	well	
7	9	city	x
11	13	city	no

The fluoride exposure series for this subject would be as follows:
florExp0=dk, florExp1=dk, florExp2=dk, florExp3=w, florExp4=w, florExp5=w, florExp6=dk, florExp7=x, florExp8=x, florExp9=x, florExp10=dk, florExp11=no, florExp12=no, florExp13= missing, florExp14= missing, florExp15= missing, florExp16=missing, florExp17= missing, florExp18= missing,
florExp19= missing

Example 3:

A subject aged 18 years, on his/her classroom exam day, with a current fluoride exposure code of "o." Further, this subject provided one row of data on previous residences as follows:

from	to	water	fl

0	4	city	m

The fluoride exposure series for this subject would be:
florExp0=m, florExp1=m, florExp2=m, florExp3=m, florExp4=m, florExp5=o, florExp6= o, florExp7= o, florExp8= o, florExp9= o, florExp10= o, florExp11= o, florExp12= o, florExp13= o, florExp14= o, florExp15= o, florExp16= o, florExp17= o, florExp18= o, florExp19= missing

Descriptive Results for Water Source and Fluoride Exposure

Descriptive data on the water sources and fluoride content regarding the current residence at the time of the survey are provided in Table S3. In all, 54.5% of the current water sources were classified as "city," 27.6% as "well," and 17.8% as "do not know." Sample distribution according to the fluoride status code for each subject's year of life is presented in Table S5.

Table 5. Counts and percentages of codes for each member (year age) in the fluoride exposure series.

SeriesElement	Quantity	dk	m	na	nb	no	o	w	x	missing
florExp0	count	2210	11	10	129	55	2050	1265	207	0
florExp0	percent	37.2	0.2	0.2	2.2	0.9	34.5	21.3	3.5	0.0
florExp1	count	1478	13	14	144	65	2426	1541	256	0
florExp1	percent	24.9	0.2	0.2	2.4	1.1	40.9	26.0	4.3	0.0
florExp2	count	1389	14	17	146	68	2467	1573	263	0
florExp2	percent	23.4	0.2	0.3	2.5	1.1	41.6	26.5	4.4	0.0
florExp3	count	1325	17	17	146	69	2498	1602	262	1
florExp3	percent	22.3	0.3	0.3	2.5	1.2	42.1	27.0	4.4	0.0
florExp4	count	1304	17	16	155	70	2504	1621	249	1
florExp4	percent	22.0	0.3	0.3	2.6	1.2	42.2	27.3	4.2	0.0
florExp5	count	1262	19	15	159	69	2531	1638	240	4
florExp5	percent	21.3	0.3	0.3	2.7	1.2	42.6	27.6	4.0	0.1
florExp6	count	1188	23	16	145	58	2472	1559	231	245
florExp6	percent	20.0	0.4	0.3	2.4	1.0	41.6	26.3	3.9	4.1
florExp7	count	1099	27	16	117	51	2265	1443	215	704
florExp7	percent	18.5	0.5	0.3	2.0	0.9	38.2	24.3	3.6	11.9
florExp8	count	980	28	16	100	42	1988	1288	214	1281
florExp8	percent	16.5	0.5	0.3	1.7	0.7	33.5	21.7	3.6	21.6
florExp9	count	856	28	14	80	32	1772	1118	213	1824
florExp9	percent	14.4	0.5	0.2	1.3	0.5	29.9	18.8	3.6	30.7
florExp10	count	761	22	8	71	25	1583	961	197	2309
florExp10	percent	12.8	0.4	0.1	1.2	0.4	26.7	16.2	3.3	38.9
florExp11	count	637	20	7	66	20	1357	788	181	2861
florExp11	percent	10.7	0.3	0.1	1.1	0.3	22.9	13.3	3.0	48.2
florExp12	count	544	22	6	57	16	1128	648	141	3375
florExp12	percent	9.2	0.4	0.1	1.0	0.3	19.0	10.9	2.4	56.9
florExp13	count	436	20	5	45	14	924	497	101	3895
florExp13	percent	7.3	0.3	0.1	0.8	0.2	15.6	8.4	1.7	65.6
florExp14	count	355	16	4	30	12	737	400	88	4295
florExp14	percent	6.0	0.3	0.1	0.5	0.2	12.4	6.7	1.5	72.3
florExp15	count	273	17	3	18	4	566	311	69	4676
florExp15	percent	4.6	0.3	0.1	0.3	0.1	9.5	5.2	1.2	78.8
florExp16	count	196	14	3	15	0	420	214	39	5036
florExp16	percent	3.3	0.2	0.1	0.3	0.0	7.1	3.6	0.7	84.8
florExp17	count	114	12	1	12	0	275	117	32	5374

florExp17	percent	1.9	0.2	0.0	0.2	0.0	4.6	2.0	0.5	90.5
florExp18	count	41	6	0	4	0	95	30	7	5754
florExp18	percent	0.7	0.1	0.0	0.1	0.0	1.6	0.5	0.1	96.9
florExp19	count	8	1	0	0	0	9	5	0	5914
florExp19	percent	0.1	0.0	0.0	0.0	0.0	0.2	0.1	0.0	99.6

Calculation of Community Water Fluoridation (CWF) Exposure Level

First, we developed a binary CWF exposure code (0/1) for each age-year using the information contained in the variables florExp0 to florExp19. If the code for "florExp" for each age is "o" or "no," the binary variable is coded as 1. If the "florExp" code is "m", "na", "nb", "x" or "w", the binary variable is coded as 0. We summed the number of 1's for this binary variable up to the age of each student and divided by their own age to calculate the percentile of the life-time CWF exposure level. If a student's "florExp" variable includes "dk" at least one time, the students were excluded from the analysis. For our analysis, we divided this percentile into four categories: "0%", "0%<, <=50%", "50%<, <100%", "100%" as CWF exposure level.
